# A 12-Gb/s Stacked Dual-Channel Interface for CMOS Image Sensor Systems

**DOI:** 10.3390/s18082709

**Published:** 2018-08-17

**Authors:** Sang-Hoon Kim, Hoon Shin, Youngkyun Jeong, June-Hee Lee, Jaehyuk Choi, Jung-Hoon Chun

**Affiliations:** 1 College of Information and Communication Engineering, Sungkyunkwan University, Suwon 16419, Korea; 2 Samsung Electronics, Hwaseong 18448, Korea; hoon.sin@samsung.com (H.S.); youngk.jeong@samsung.com (Y.J.); junehee.lee@samsung.com (J.-H.L.)

**Keywords:** dual-channel, transceiver, charge-recycling, shared CDR, CMOS Image Sensor (CIS) System, stacked driver

## Abstract

We propose a dual-channel interface architecture that allocates high and low transition-density bit streams to two separate channels. The transmitter utilizes the stacked drivers with charge-recycling to reduce the power consumption. The direct current (DC)-coupled receiver front-end circuits manage the common-mode level variations and compensate for the channel loss. The tracked oversampling clock and data recovery (CDR), which realizes fast lock acquisition below 1 baud period and low logic latency, is shared by the two channels. Fabricated in a 65-nm low-power complementary metal-oxide semiconductor (CMOS) technology, the dual-channel transceiver achieves 12-Gb/s data rate while the transmitter consumes 20.43 mW from a 1.2-V power supply.

## 1. Introduction

In CMOS image sensor (CIS) systems, as the pixel resolution and the frame rate increases, the data transmission bandwidth between the sensor and image signal processor (ISP) continuously increases. As the speed of the interface increases, the increase in power consumption can not be avoided and this limits the available lifetime of the devices for battery-powered applications. In addition, especially in the case of CIS systems, the large power dissipation from the interface circuit near a sensor array causes local heating on the sensor side, resulting in image degradation due to an increase in the dark-current. Therefore, the power efficiency is one of the most important indicators for evaluating the CIS interface circuits on the sensor side.

Several standardized interfaces such as D-PHY and C-PHY from the mobile industry processor interface (MIPI) alliance can support required data rates for a CIS system. First, the MIPI D-PHY v1.2 (MIPI Alliance, Piscataway, NJ, USA) [1] can support 2.5-Gb/s peak data rate in high-speed (HS) mode; 10-Gb/s data transmission rate can be achieved with four differential data lanes and additional two pins to forward the clock (the latest version of MIPI D-PHY, v2.0 (MIPI Alliance, Piscataway, NJ, USA), can support a maximum of 4.5-Gb/s data rate per lane). Since the D-PHY requires additional lanes for clock forwarding, the effective data rate per channel must be reduced. The MIPI C-PHY [1,2] has improved throughput performance compared to the D-PHY. The C-PHY transmits the 3-phase coded data with clock embedding through the 3-wire lanes. The 1-symbol data of 3-bits can have five possible transitions by flip/rotate/polarity, and can transmit and receive 7-symbol words to represent 16-bit data. The effective bandwidth of the C-PHY is 11.4 Gb/s with six embedded clock and data lanes [3]. However, the C-PHY requires rather complicated symbol encoding, and a unique clock and data recovery (CDR) algorithm referred to as a “triggered eye concept”. On the other hand, the scalable low voltage signaling embedded clock (SLVS-EC) still uses the conventional non-return-to-zero (NRZ) signaling with clock embedding. Therefore, the clock and data recovery is relatively easy compared with C-PHY. In addition, the SLVS-EC can reduce the power consumption by lowering the voltage swing of the output driver, but it requires an additional power regulator to set the output voltage swing level.

In this paper, we propose a power-efficient dual-channel CIS interface architecture using the transition characteristics inherent to image signals. The proposed architecture does not require the complex symbol encoding unlike C-PHY. The transmitter utilizes the stacked drivers with charge-recycling to enhance the power efficiency. This paper is organized as follows: Section 2 describes how we exploited the characteristics of image signals in the proposed dual-channel architecture. In Section 3, the details of the implemented circuits including the stacked driver of transmitter, the receiver front-end circuits, the low-power data path, and the CDR circuits are presented. The experimental results are presented in Section 4, followed by the conclusions in Section 5.

## 2. Backgrounds and Proposed Architecture

**Figure 1:**
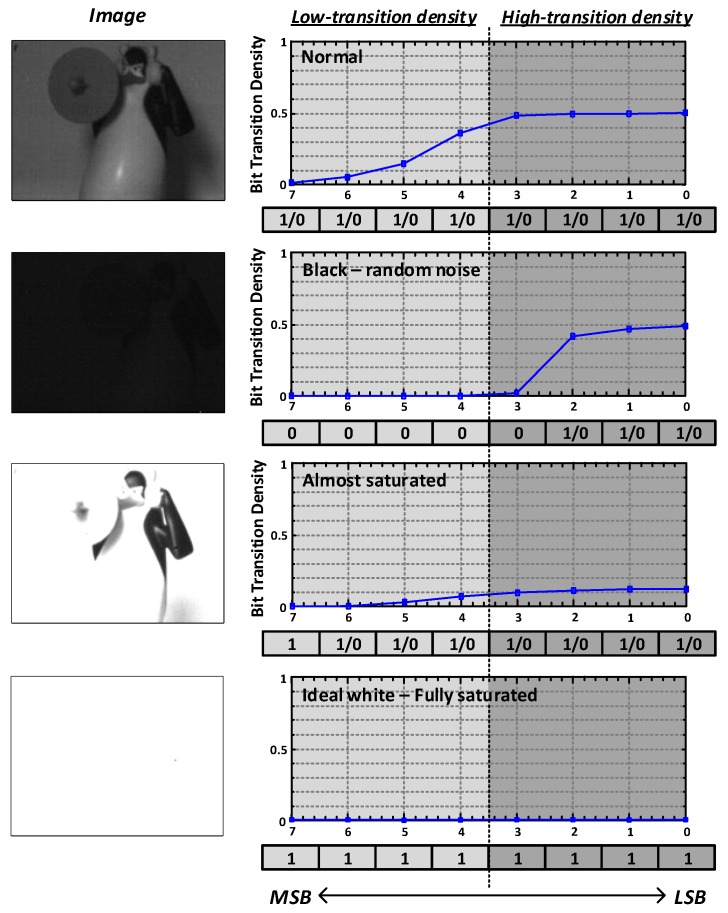
Bit transition behavior of ADC output data with various luminance conditions.

The raw image data which is the digital output of the CMOS image sensor has unique transition characteristics. Figure 1 shows the images of the same object taken several times with different illuminance, and pixel-to-pixel transition density of each bit position of the 8-bit analog-to-digital converters’ (ADCs’) output. In general, the transition density of the image signal is higher as the bit position is closer to the least significant bit (LSB). As shown in Figure 1, for the image taken at the normal illuminance, the transition density on the LSB side is higher than that on the most significant bit (MSB) side. Almost all bits of the black image are zero, but the three bits on the LSB side still toggle because of random noise. For the nearly white image that are almost saturated by strong light, toggling still exists in the LSB.

**Figure 2:**
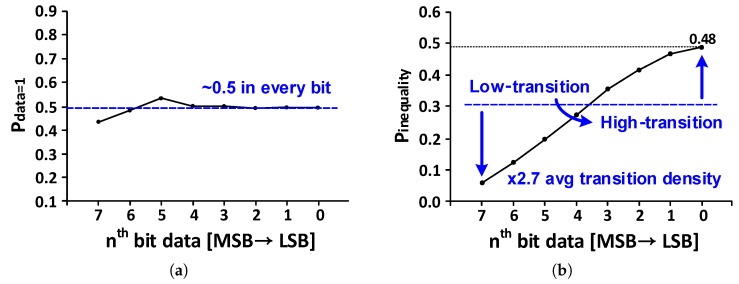
Transition characteristics of one hundred test image results (**a**) $P_{data=1}$; (**b**) $P_{inequality}$.

Figure 2 shows the transition characteristics of one hundred test images from the public image database [4,5]. As shown in Figure 2a, the probability that each bit has value of “1” ($P_{data=1}$) is kept around 0.5. However, the transition probability that the neighboring pixels have different values ($P_{inequality}$) is not the same for each bit as shown in Figure 2b. The pixel-to-pixel transition density is the highest at 48% in the LSB and decreases to 6% in the MSB. When the 8-bit data is divided into the 4-bit data closer to the MSB and the 4-bit data closer to the LSB, the average transition density on the LSB side is 2.7 times higher than that on the MSB side. That is, all data bits have almost the same toggling density for the whole image; however, for adjacent data bits, the toggling density is high on the LSB side. Based on this observation, 8-bit image signals can be classified into two groups: low-transition density (LTD) and high-transition density (HTD) data as shown in Figure 1. The lower 4-bit data (LSB—4th bit) is classified as HTD data and the upper 4-bit data (5th bit—MSB) is classified as LTD data. In general, when recovering a clock from data, additional encodings such as 8b10b or 10b12b are needed because it is important that the data transition density is retained above a certain level. However, using the unique transition characteristic of the image signals described above, the sufficient transition density for the CDR operation can be obtained without additional data encoding. Therefore, we propose a dual-channel transceiver architecture in which the clock on the receiver side is recovered by the CDR circuit located only on the HTD channel.

**Figure 3:**
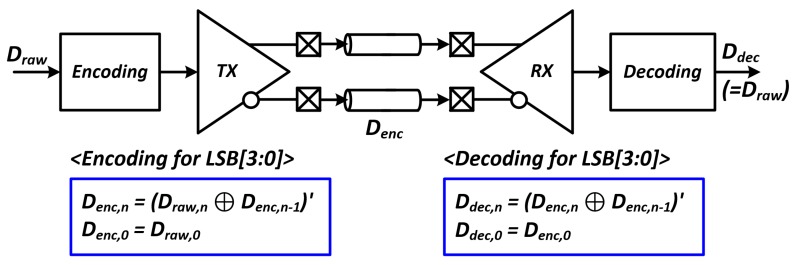
Simple coding logic for the transition issue on fully saturated white images.

Unfortunately, we cannot completely exclude the data encoding. As shown at the bottom of Figure 1, the fully saturated white image has zero transition density at every bit position. Even in the almost saturated image, the bit transition density on the LSB side is significantly lower than that of the non-saturated normal images. However, to insert sufficient transitions for the saturated images, we can use simple but efficient coding schemes for four LSBs as shown in Figure 3, which do not cause the reduction in effective bandwidth. In this work, we used a simple encoding scheme that inverts the next symbol when the saturated symbol is detected:

(1) $$\begin{array}{cc} D_{enc,n} & ={\left(D_{raw,n}⊕D_{enc,n-1}\right)}^{\prime }, \end{array}$$

(2) $$\begin{array}{cc} D_{enc,0} & =D_{raw,0}, \end{array}$$

(3) $$\begin{array}{cc} D_{dec,n} & ={\left(D_{enc,n}⊕D_{enc,n-1}\right)}^{\prime }, \end{array}$$

(4) $$\begin{array}{cc} D_{dec,0} & =D_{enc,0}. \end{array}$$

The simple logic equation for encoding in a transmitter and decoding in a receiver are respectively shown in Equations (1)–(4), respectively. $D_{raw,n}$ denotes the *n*-th CIS raw data. $D_{enc,n}$ denotes the *n*-th encoded data, and $D_{dec,n}$ denotes the n-th decoded (recovered) data.

**Figure 4:**
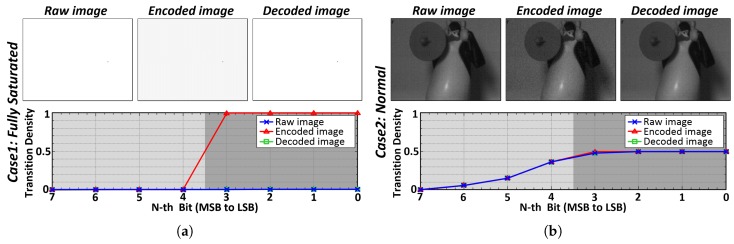
Raw, encoded, and decoded images and their bit transition densities: (**a**) fully saturated image; (**b**) normal image.

Figure 4 shows the transition densities of raw, encoded, and decoded data when logic is applied to only the LSB side. When the image is fully saturated in Figure 4a, the LSB side of the CIS raw data is encoded by the encoder of the transmitter and the transition density is increased. On the receiver side, the decoder restores the data and produces the same data as the raw data. As shown in Figure 4b, when the normal image is encoded the transition density is slightly changed and the encoded image is weakly corrupted. However, when the data is decoded, the image is restored identical with the raw image.

**Figure 5:**
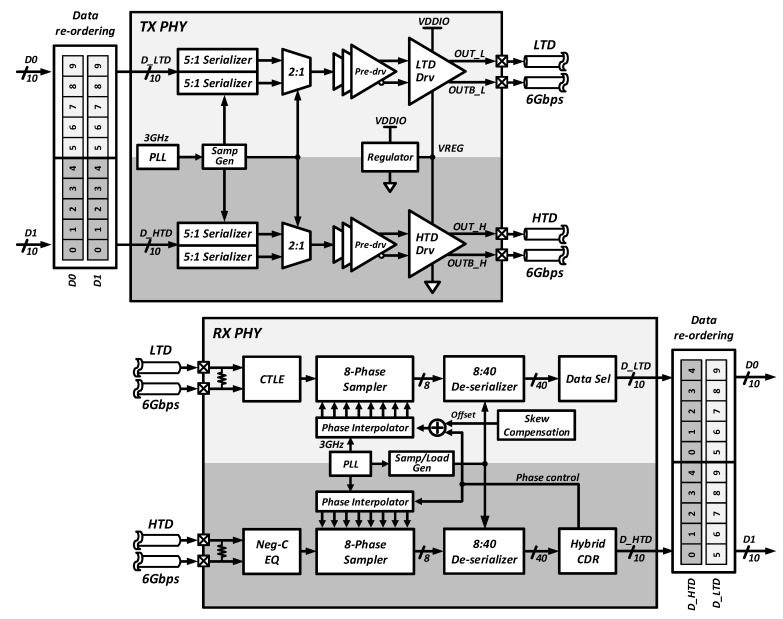
Overall PHY architecture of the proposed dual-channel transceiver.

Figure 5 represents the overall PHY architecture of the proposed dual-channel transceiver. In this architecture, two sets of 10-bit data (D0, D1) are rearranged into 10-bit LTD data (D_LTD) and HTD data (D_HTD), and transmitted through the dual channels. First, two 10-bit CIS raw data are re-ordered in the transmitter.

The upper five bits and the lower five bits of the 10-bit are routed to the LTD and HTD paths, respectively. The transmitted data using the LTD and HTD driver are received through two different front-end circuits because of different input voltage levels. On the receiver side, the clock is restored by the HTD data transmitted to the bottom-side channel, and the LTD path uses the same clock because rich clock-transition density of the HTD path can reduce the clock jitter.

## 3. Circuit Details

As mentioned before, the proposed transceiver transmits and receives the allocated HTD and LTD data to share the CDR that is located only in the HTD path, and all the circuits are designed to support the dual-channel architecture. Owing to the dual-channel architecture, the clocking circuits such as the phase-locked loop (PLL), clock dividers, and the sampling signal generator (SampGen) for the data paths are shared between the two separate channels.

Another distinctive feature of the proposed transceiver is a differential stacked driver using a charge-recycling technique that reduces the static power by half. The transmitter consists of a data path, clocking circuits, and an output driver with a simple regulator. The voltage-mode drivers for the two separate data paths are stacked from ground to supply, and the middle node between the two drivers is regulated by a simple push–pull regulator.

The receiver consists of an on-die termination (ODT) circuit, continuous-time linear equalizer (CTLE), and data path including 8-phase samplers and de-serializers, and phase interpolators (PI) to recover the clock and data. In the receiver, two different CTLEs are separately used in the HTD and LTD paths in order to cover the different input common-mode voltage levels due to the use of the stacked driver in the transmitter. The equalized signals are oversampled by an 8-phase sampler and the clock phases are controlled by the phase interpolater (PI). The hybrid CDR [6] consisting of the oversampling phase detector (OSPD) and the bang-bang phase detector (BBPD) is located only in the HTD path and controls the phase interpolator. In the LTD path, the data selector block (DataSel) detects the data and edge positions and selects the proper 10 bits out of 40 input bits. The PI of the LTD path is also controlled by the CDR in the HTD path, and the possible timing skew between the two channels can be compensated by the skew compensation block. In addition, the CDR circuit realizes fast lock acquisition and low logic latency to meet the requirements of MIPI low latency interface (LLI) specifications. The circuit details are as follows:

(5)HLS,NC(s)≃Gm·(RD∥ZOUT,NC)(6)≃gm·[RD∥{-(1gmN+12sCN)∥1sCL}],withGm≃gm,(7)≃gm·2RDCNs+gmNRD(2CNRDCL)s2+(gmNRDCL-2gmNCNRD+2CN)s+gmN,

(8) $$\begin{array}{cc} \;w_{z} & =g_{mN}/2C_{N}. \end{array}$$

### 3.1. Charge-Recycling Differential Transmitter Driver

**Figure 6:**
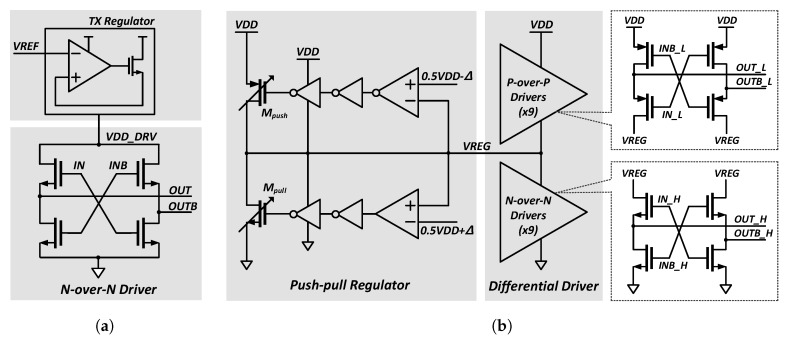
Schematic diagrams of (**a**) N-over-N type transmitter with a power supply regulator and (**b**) charge-recycling differential stacked driver with a push–pull regulator.

We adopted the segmented voltage-mode driver because it is suitable for low-swing, low-power interfaces. The number of turned-on segments can be adjusted to match the output impedance. Figure 6a shows a conventional low-swing N-over-N voltage-mode driver with a supply voltage regulator. The voltage-mode driver can save the dynamic power owing to the scaled low-voltage swing by the regulator. However, a substantial portion of static power is wasted by the linear regulator on the top of the output driver. Therefore, to save the static power used in the linear regulator, we replaced the linear regulator with a differential P-over-P driver as shown in Figure 6b. The additional push–pull type regulator regulates the middle-node voltage, VREG, as 0.5·VDD. Since the proposed stacked driver consists of the two “differential” drivers, the constant static current flows through the P-over-P driver and the N-over-N driver. Therefore, a low-bandwidth regulator of [7] is not required to compensate for the static current variation. The relatively small push–pull regulator compensates the unbalanced dynamic power between the top and bottom channels, and thus, keeps VREG between “0.5·VDD+Δ” and “0.5·VDD-Δ”. The push–pull regulator consists of $M_{push}$, $M_{pull}$ and two comparators that compare VREG with the reference voltage levels. The reference voltages of a pair of comparators are “0.5·VDD-Δ” and “0.5·VDD+Δ”, respectively.

**Figure 7:**
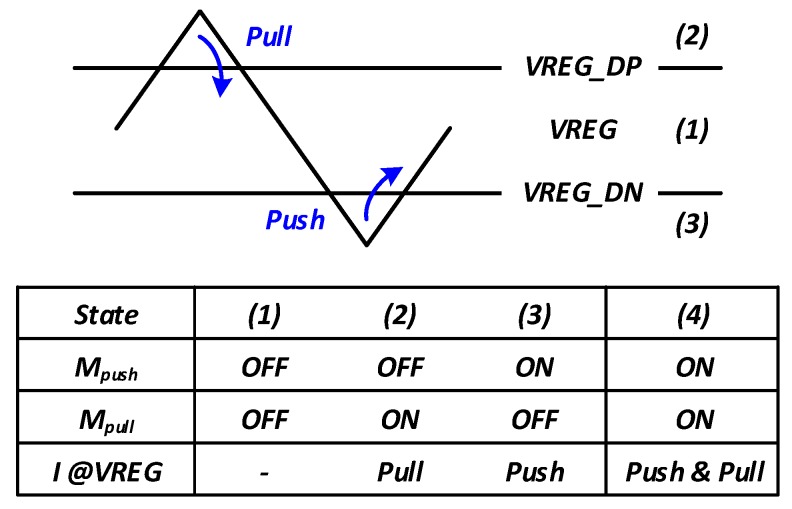
Operating principles of push–pull regulator.

Figure 7 shows the operating principle of the push–pull regulator. If VREG is between “0.5·VDD+Δ” and “0.5·VDD-Δ” as in state (1), both $M_{push}$ and $M_{pull}$ transistors do not operate, and the regulator maintains the same state as before. However, if VREG is greater than “0.5·VDD+Δ” as in state (2), the $M_{pull}$ transistor of the regulator is activated, pulling down the VREG potential below “0.5·VDD+Δ”. In contrast, in state (3), the $M_{push}$ transistor of the regulator operates and pushes the current from VDD to VREG.

### 3.2. Receiver Front-End Equalizers

**Figure 8:**
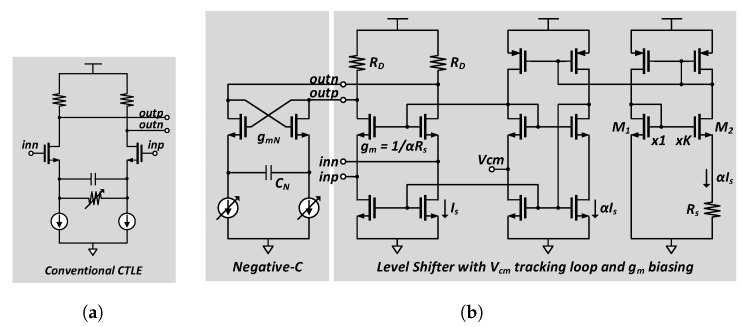
Schematic of (**a**) conventional CTLE in LTD channel; and (**b**) one-stage level shifter with the negative-C circuit in HTD channel.

As mentioned before, the two input common-mode voltages of the dual-channels in the receiver are different since the stacked driver is used in the transmitter for charge recycling. For the LTD channel which receives the data through the upper side of the stacked driver, the input common-mode voltage on the receiver side is as high as 0.75·VDD. Therefore, for the LTD channel, we used the conventional analog continuous-time linear equalizer (CTLE), an NMOS common-source amplifier with source degeneration as shown in Figure 8a. However, in the HTD channel, data is transmitted by the lower side of the stacked driver; therefore, the input common-mode voltage of the receiver is lowered to 0.25·VDD. To use the CTLE of the same structure in the HTD channel, we need an additional level-up shifter. Using the additional level shifter causes timing skew between the two channels, which is not desirable because the HTD and LTD receivers share a CDR in the HTD path. An attractive alternative is a level shifter with an integrated negative-C circuit shown in Figure 8b.

The level shifter is a simple common-gate amplifier, and its output common-mode voltage is shifted up to an appropriate voltage level to drive the sampler in the next stage. To prevent the voltage gain variation of the level shifter due to changes in the surrounding environment, the constant-$g_{m}$ biasing circuit [8] is used. Owing to the addition of the negative-C circuit, this level-up shifter has the peaking gain at the nyquist frequency, thereby acting as an analog equalizer in the LTD channel. The overall voltage gain of the circuit in Figure 8b can be derived as Equations (5)–(7). In those equations, $Z_{OUT,NC}$ denotes the output impedance of the negative-C circuit, and $C_{L}$ denotes the load capacitance at the output node. A zero is located at $g_{mN}/2C_{N}$ as shown in Equation (8), where $g_{mN}$ and $C_{N}$ are the $g_{m}$ and capacitance of the negative-C circuit, respectively. Adjusting the $g_{m}$ of the input transistor, we can adjust the zero frequency. The implemented level shifter has the peaking gain of 6 dB at 3 GHz with a default setting. By using the “single-stage” level-up shifter with the equalizer, the timing skew between the two channels can be minimized.

The receiver front-end of the LTD and HTD channels can be digitally adjusted. The CTLE in the LTD channel can be controlled by a 4-bit resistor array of the source degeneration part. The proposed level-up shifter with the negative-C circuit in the HTD channel can be controlled by a 4-bit current DAC. The timing skew between the equalizer of the LTD channel and the HTD channel ranges from −8.3 ps to 3.7 ps in 9-corner simulations.

### 3.3. Low-Power Data Path

**Figure 9:**
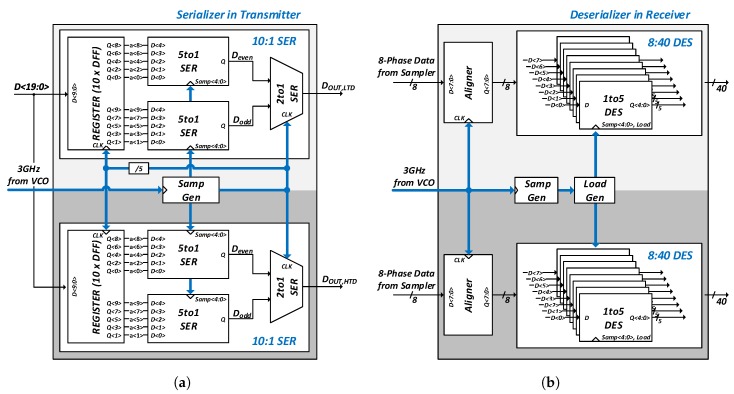
Block diagram of data paths: (**a**) serializer in transmitter (TX) and (**b**) de-serializer in receiver (RX).

**Figure 10:**
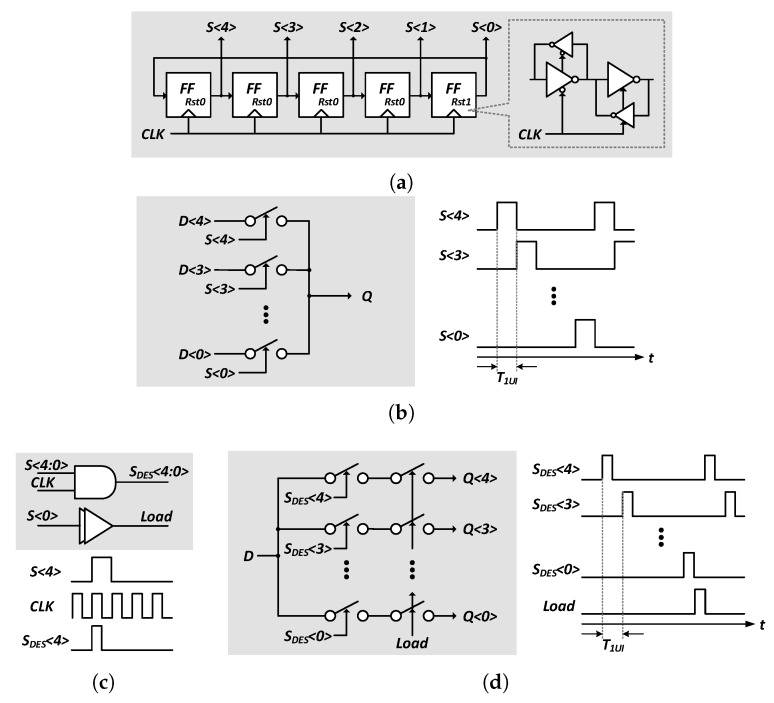
(**a**) block diagram of SampGen block; the block diagram and timing diagram of (**b**) 5-to-1 serializer in the transmitter; (**c**) LoadGen block; and (**d**) 1-to-5 de-serializer in the receiver.

As shown in Figure 9a, the serializer of the transmitter receives 20-bit data and distributes 10 bits of the MSB and LSB to the LTD side and HTD side, respectively. The sampling signal is used to sample and serialize 10-bit parallel data from two 5-to-1 serializers on each channel. The even and odd data are converted to a single data stream by a binary serializer and transferred to the pre-driver. The de-serializer of the receiver is shown in Figure 9b. The 8-phase data from the sampler are aligned first and fed to the following 1-to-5 de-serializers. Figure 10 shows the SampGen block shared by both channels. As shown in Figure 10a, five repeating pulses with a pulse width of a clock period are generated by a flip-flop chain with the last output connected to the first input. These sampling signals are sent to the 5-to-1 serializer of the transmitter and the LoadGen block of the receiver, respectively, as shown in Figure 10b,c. The LoadGen block generates the sampling and load signals and send them to the 1-to-5 deserializer as described in Figure 10d. The parallelized 40-bit data are used for clock and data recovery in a bang-bang phase detector (BBPD) and the oversampling phase detector (OSPD) of the CDR.

### 3.4. Low-Power and Fast-Acquisition All-Digital CDR

**Figure 11:**
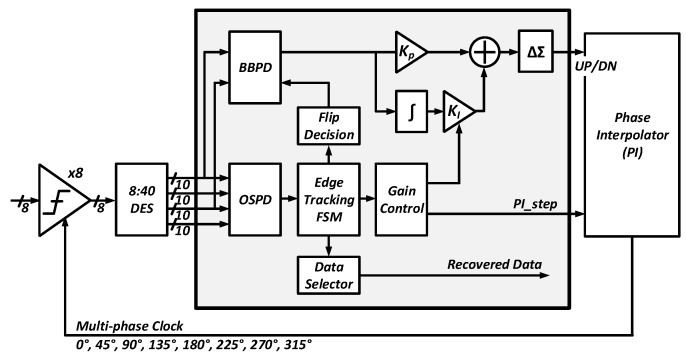
Block diagram of the hybrid-CDR.

Figure 11 shows a block diagram of the digital CDR. Unlike the conventional bang-bang phase detector (BBPD)-based CDR, a hybrid structure of BBPD and oversampling phase detector (OSPD) is used. In BBPD, 0${}^{\circ }$ and 180${}^{\circ }$ phase clocks are placed at the center of the data. The OSPD tracks the edge of data using 8-phase clocks. In the conventional BBPD-based CDR, if the detection range exceeds $1UI_{p2p}$, it is impossible to recover the data since the lead and lag signals are inverted. To solve this problem, the edge-tracking finite-state machine (ET-FSM) detects the position of the edge using the output of the OSPD and sets the state. Depending on the ET-FSM’s state, the gain of the integral path ($K_{I}$) or the interval of the steps in the phase interpolator (PI) is adjusted to expand the detection range up to $2.5UI_{p2p}$, while the detection range of the conventional BBPD-based CDR is less than $1UI_{p2p}$. The recovered data is exported by the data selector using the edge position and state information. Using the data selector and ET-FSM instead of the elastic FIFO, a logic latency of less than 2-baud periods was obtained. The detailed operating principle of the proposed CDR is described in [6].

## 4. Experimental Results

**Figure 12:**
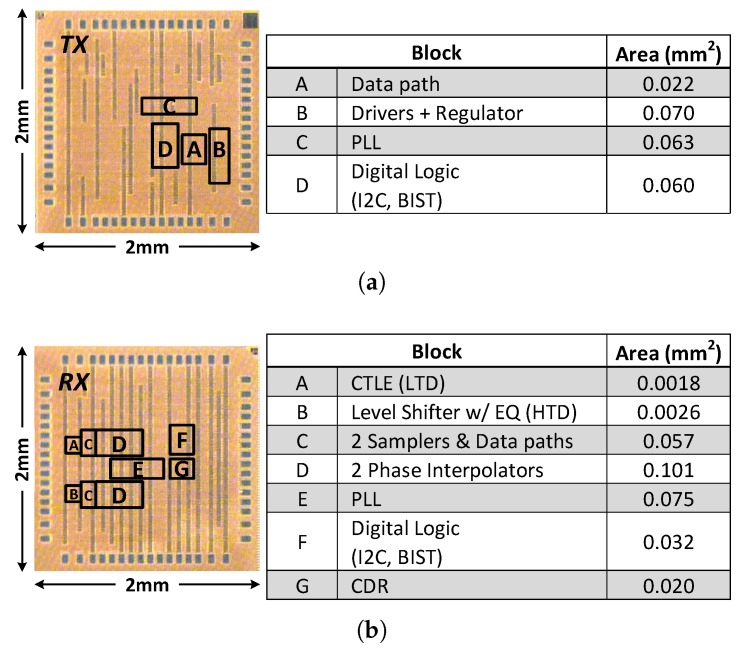
Die microphotograph and area breakdown of the transceiver fabricated in a 65-nm CMOS technology: (**a**) TX; and (**b**) RX.

The proposed transceiver circuits are implemented in a 65-nm low-power CMOS process with a 1.2-V supply. Figure 12a,b show the die microphotographs of the transmitter and receiver, respectively. The total area of each chip is 2 mm × 2 mm, and the transmitter and receiver cores occupy 680 μm × 650 μm, and 1100 μm × 570 μm, respectively. Each of the LTD and HTD channels provides 6-Gb/s data rate, so the aggregate bandwidth of the dual-channel is 12 Gb/s.

**Figure 13:**
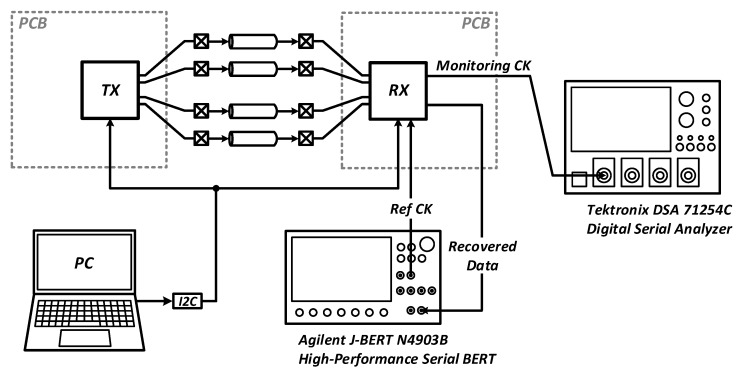
Measurement setup.

Figure 13 shows the test setup for the transceiver. The PRBS generator of the built-in self-test (BIST) block in the transmitter creates parallel data that are serialized and transmitted to the receiver. The error-free operation of the data path is confirmed by the BIST circuits embedded in the transmitter and receiver, respectively.

**Figure 14:**
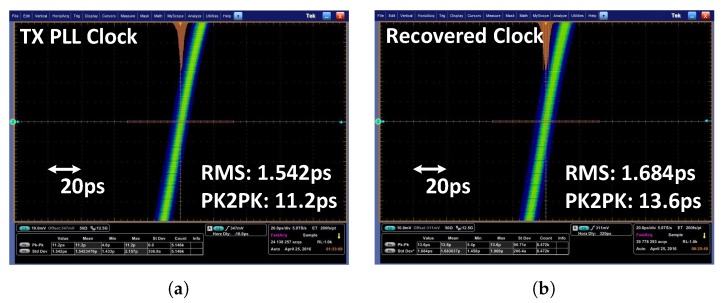
Jitter performance: (**a**) transmitter PLL clock; and (**b**) receiver recovered clock.

Figure 14 shows the jitter performance of the transmitter PLL clock and the recovered clock in the receiver. When the clock frequency is 3 GHz, the root mean square (RMS) and peak-to-peak jitters of the transmitter PLL output clock are 1.542 ps and 11.2 ps, respectively. The recovered clock of the receiver has 1.684-ps RMS jitter and 13.6-ps peak-to-peak jitter when a normal image data set is received and recovered.

**Figure 15:**
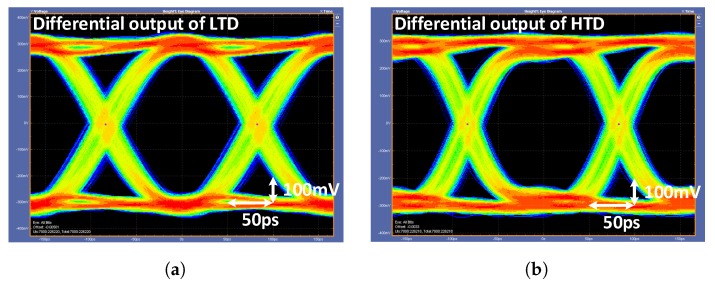
Eye diagram of the transmitter output: differential output of (**a**) LTD and (**b**) HTD.

**Figure 16:**
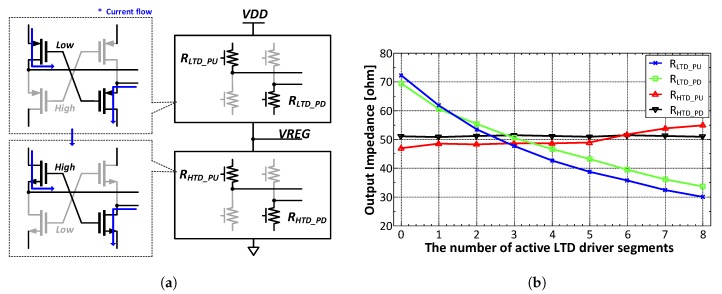
(**a**) simplified driver resistance model, and (**b**) resistance of the driver’s transistor vs. the number of active LTD driver segments.

The measured differential eye diagrams of the LTD and HTD driver outputs are shown in Figure 15a,b, respectively. The differential eye diagrams were measured with the Tektronix P7313SMA differential probes (Beaverton, OR, USA). We can calibrate the output impedance of each driver by controlling the number of active segments. Figure 16a shows a simplified resistance model of the driver. Figure 16b shows how the pull-up and pull-down resistances of each driver are changed when the number of active P-over-P segments is changed. In this measurement setup, the number of active N-over-N segments is fixed. It is noteworthy that the N-over-N driver’s impedance remain fixed because the regulator keeps VREG as 0.5·VDD. If the regulator is turned off, the pull-up resistance of the N-over-N driver ($R_{HTD\_PU}$) varies from 35 Ω to 70 Ω.

**Figure 17:**
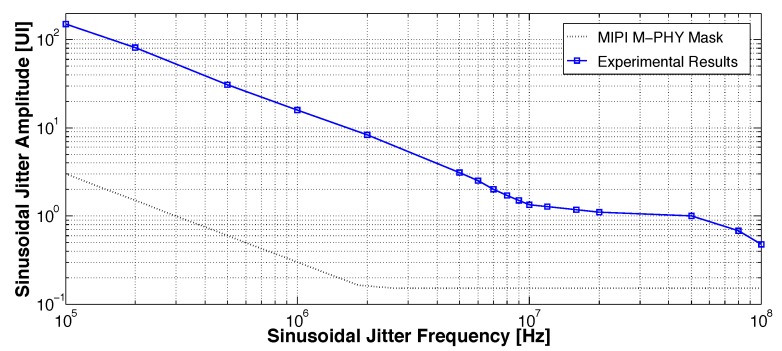
Jitter tolerance.

Figure 17 compares the jitter tolerance test results of the proposed CDR with a sinusoidal jitter mask of MIPI M-PHY HS-G1. The proposed CDR has sufficient jitter tolerance margin while the corner frequency is approximately 10 MHz.

**Figure 18:**
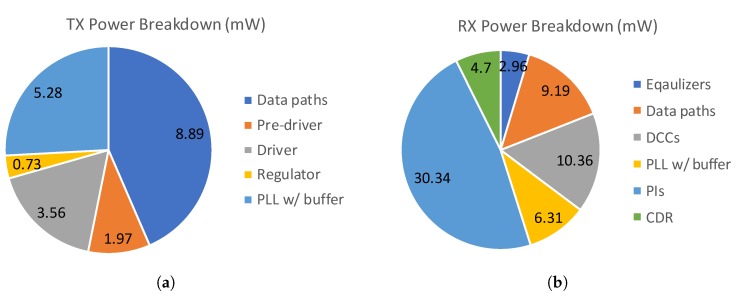
Power breakdown: (**a**) TX and (**b**) RX.

Figure 18 shows the power breakdown for the transmitter and receiver chips at 12 Gb/s. The output stage of the transmitter, which consists of a stacked driver and regulator, consumes 4.29 mW with 12-Gb/s output data with 600-mV${}_{\mathrm{ppd}}$ swing.

Table 1 shows a comparison with previous work. FoM1 reflects the process and the output swing of the transmitter on the general FoM. Based on the results of Table 1, we can conclude that the proposed transceiver has superior energy efficiency.

## 5. Conclusions

We demonstrated a power-efficient dual-channel transceiver for a CMOS image sensor system using a 65 nm low-power CMOS (LP-CMOS) technology with a 1.2 V supply. Using the unique transition characteristic of image signals, we could rearrange the raw data into high and low transition-density data streams, and a total 12-Gb/s data transmission bandwidth with good signal integrity was achieved. In addition, the proposed architecture does not require complex data encoding to recover the clock and data through the data rearrangement, and does not sacrifice effective bandwidth. Owing to the charge-recycling stacked driver and low-power data path, we could significantly improve power efficiency. The equalizer with level-up shifting in the HTD path could resolve the different input common-mode problem and minimize the timing skew between the HTD and LTD channels. The tracked oversampling CDR was successfully integrated with the proposed architecture, and the skew compensation circuitry in the CDR could solve any possible additional timing skews.

## Figures and Tables

**Table 1:** Performance summary and comparison.

References	JSSC12 [9]	JSSC13 [10]	JSSC16 [11]		JSSC15 [12]		This work
	A. Amirkhany	J. W. Poulton	B. Dehlaghi		T. O. Dickson		
Process	40-nm	28-nm	28-nm FD-SOI		32-nm SOI		65-nm
Supply [V]	0.9/1/1 (R/A/IO)	0.9	1		1		1.2
Data rate [Gb/s]	12.8	20	18	20	12		12
Signaling	SE VM	SE GRS	SE VM		Diff CM		Diff VM
Application	Chip-to-chip	Chip-to-chip (On-package)	Die-to-die		Chip-to-chip		Chip-to-chip
Equalization	TX FIR RX CTLE+DFE	X	TX passive		RX CTLE+1-DFE		RX CTLE
Channel loss [dB] @ Nyquist	10	1	13.5 (7.7) *	10.7 (5.9) *	3	14	6
TX swing [Vpp]	0.6	0.15	-		0.4 (diff)		0.6 (diff)
FoM [pJ/bit] **	2.15	0.255	0.32	0.3	1.08	1.58	2.275
FoM1 *** [FoM/nm${}^{2}$/V]	2.240 M	2.168 M	-	-	2.636 M	3.856 M	0.897 M

* Relative to DC; ** Excluding the clocking circuits’ power consumption, based on the reported power breakdown; *** FoM1 = FoM / (Process${}^{2}$[nm${}^{2}$] × TX swing[V]). Abbreviation: Fully depleted silicon on insulator (FD-SOI), Single-ended (SE), Differential (Diff), Voltage-mode (VM), Current-mode (CM), Ground-referenced signaling (GRS), Finite impulse response (FIR), Continuous-time linear equalizer (CTLE), Decision feedback equalizer (DFE).
